# Existence and significance of viral nonreplicative RNA recombination

**DOI:** 10.1371/journal.pbio.3002216

**Published:** 2023-07-25

**Authors:** Vadim I. Agol

**Affiliations:** A.N. Belozersky Institute of Physical-Chemical Biology, M.V. Lomonosov Moscow State University, Moscow, Russia

## Abstract

In response to a recent article, this Formal Comment discusses nonreplicative joining of fragments of viral RNAs, a class of reactions which might be widespread in nature, contributing to conservation and evolution not only of viruses but of cellular organisms as well.

Recombination between genomes of RNA-viruses is a key actor in their micro- and macroevolution as well as in repair after genetic injuries [1,2]. Replicative and nonreplicative forms of recombination were recognized in these viruses; the former is accomplished by the viral RNA-dependent RNA polymerase (RdRp), whereas the latter is not. However, Kim and colleagues, in a recent publication in this journal [3], negated the existence of the latter mode on the basis of their discovery of a novel, noncanonical IRES-independent RNA translation in some IRES-containing viruses. They claimed that “… all of the studies supporting nonreplicative recombination … employed RNA transfection of RNAs capable of expressing the RdRp.” Although their paper indeed expands our understanding of translation initiation, the conclusion about the nonexistence of nonreplicative recombination in RNA-viruses cannot be considered accurate. Such mechanism is a real phenomenon, as convincingly shown by our laboratory and others. Importantly, a related class of reactions might be widespread in the nature, contributing to the evolution not only of viruses but of cellular organisms as well. Hence, it is appropriate to briefly summarize here the current knowledge on this process.

## The direct evidence of nonreplicative recombination in RNA-viruses

Originally, we demonstrated the existence of nonreplicative recombination in animal RNA viruses by using different pairs of fragments of the poliovirus genome [4]. The 5′-fragments contained a portion of the 5′-untranslated region (5′ UTR), harboring the essential translational element (IRES), but with the protein-coding sequences removed. Their partners were IRES-less, and thus non-translatable fragments of the viral RNA. In each pair, the partners overlapped within the hypervariable region located between the IRES and the start codon of the viral polyprotein. To enable mapping recombination sites, some substitutions were introduced into these overlapping segments of the both partners. Numerous viable, mostly nonhomologous, recombinants with crossovers within this promiscuous region were detected in the transfected cells. Recombination took place between any pairs of nucleotides (albeit with some preferences) [4,5] but the crossover sites were distributed not evenly, demonstrating several hot spots. Since neither of the fragments by itself could generate the RdRp, we concluded that recombination was nonreplicative.

However, in the original [4] and a subsequent [6] (not cited by Kim and colleagues) papers, we mentioned a possibility, although then seemingly not particularly likely, that a low level of translation of the apparently untranslatable 3′-fragments could have occurred and that the trace amounts of thus-formed viral RdRp might have generated recombinants by the canonical replicative mode. Therefore, we sought to obtain some direct evidence of the existence of nonreplicative RNA recombination and succeeded in it.

In these experiments [6], none of the recombination partners harbored the full RdRp-encoding sequence, but rather, only nonfunctional portions thereof. Because upon transfection, different pairs of such fragments also generated a variety of recombinants, we could safely conclude that the recombination in this case was unquestionably nonreplicative.

These recombinants were most likely formed by different mechanisms. Several pairs of the partners were represented by the full poliovirus RNA molecules with single nicks between neighboring nucleotides in different parts of the RdRp-encoding sequence. When such 5′- and 3′-partners contained 3′-phosphate and 5′-OH termini, respectively, efficient precise ligation was observed, yielding viable viruses. The partners with overlapping (>170 nt-long) sequences within the RdRp gene also generated homologous recombinants. Depending on the forms of their to-be-joined nucleotides (phosphorylated, dephosphorylated or triphosphorylated in the case of the 3′-partner), the chimeric genomes contained either one of the partners in its entirety or fragments of both partners. The exact reconstitution of the viral genome in the overwhelming majority of these recombinants most likely resulted from their selective survival because nonhomologous joining (likely also occurring) would predominantly generate nonviable genomes. The location of the crossover sites could be mapped only in a few cases due to the nearly complete identity of the overlapping sequences. However, by using fragments of heterotypic poliovirus RNA (with overlaps in each pair having sufficiently different nucleotide sequences but encoding nearly identical polypeptides), we demonstrated that the crossovers could occur also in many places and again with a few hot spots [5]. With specially designed partner fragments derived from poliovirus and coxsackievirus RNAs, we observed acquisition of such novelties as conversion of the noncoding sequence into a coding one (creation of a “leader” protein) and vice versa conversion of a protein-coding sequence into a noncoding one [5].

It could not be ruled out that some homologous recombinants were secondary recombinants arising through additional RdRp-dependent steps but the primary covalent joining of the partners unambiguously occurred without the participation of this enzyme, that is, nonreplicatively.

Some similarities between the effects of the structures of terminal nucleotides of recombinational partners on the efficiency and character of crossovers in our experiments targeted to the 5′ UTR [4] and RdRp [5,6] suggests that at least the majority of recombination events in the first system were also nonreplicative.

## General remarks

The existence of nonreplicative recombination in different RNA viruses was reported by several other labs ([2] and references therein) although the number of such examples is so far limited. At least in part, this could be due to the infeasibility, in some viral systems, of the conditions required for nonreplicative crossovers (e.g., timely co-localization of participants in the appropriate forms). Notably, however, there are no indications that nonreplicative RNA recombination requires any solely virus-specific mechanisms. Thus, it can be expected to occur widely in the biological world, and therefore, it seems appropriate to briefly summarize what we now know and do not know about this class of events.

Four types of nonreplicative covalent joining of RNA fragments have been demonstrated: (1) direct ligation of the 3′- and 5′-terminal nucleotides of the partner fragments; (2) invasion of the 3′-end of one partner into the internal sequence of the other one; (3) similar invasion of the 5′-end; (4) recombination between internal nucleotides of the 2 partners. The feasibility and efficiency of such reactions depend on some structural features such as the nature of the nucleotides to-be-joined and of the secondary and tertiary structure around the recombination site. These requirements, although far from being well characterized, do not appear to be extremely strong, suggesting that nonreplicative recombination could be relatively common.

The reactions involved in nonreplicative RNA recombination are catalyzed by cellular enzymes that remain unidentified. Among potential candidates are ligases, endo- and exonucleases and enzymes catalyzing transesterification. Some other actors such as ribozyme-like structures, cannot be excluded either. An important requirement is the timely co-localization of participants of the process.

The nonreplicative RNA recombination most likely generates a variety of nonfunctional “dead” products. The viable chimeric viral genomes should be infectious, replicable (by exploiting the newly formed or helper’s RdRp), and able to withstand the competition with other co-replicating viruses (including their own parents). Putative recombinants between cellular RNAs, in order to persist through generations, should be reverse-transcribed and integrated into the genomic DNA. Due to all of the aforementioned complications, nonreplicative RNA recombination generates functional viral genomes far less frequently than replicative recombination. However, the promiscuity of nonreplicative recombination implies that it could be a widespread contributor to rare, but important, biological events [5–8]. In particular, nonreplicative recombination might be responsible for some of the numerous known [9,10] (and likely many more unknown) examples of horizontal transfer of genetic information between viruses and between viruses and cells, resulting in the acquisition of novel proteins and functional RNA elements. This process potentially could be used for genetic engineering.
